# Comparison of Clinical Outcomes between Salvage and Elective Thoracic Endovascular Aortic Repair in Patients with Advanced Esophageal Cancer with Aortic Invasion: A Retrospective Cohort Study

**DOI:** 10.3390/biomedicines9121889

**Published:** 2021-12-12

**Authors:** Sian-Han Lin, Jang-Ming Lee, I-Hui Wu

**Affiliations:** 1 Department of Surgery, National Taiwan University Hospital, Taipei 10002, Taiwan; 115727@ntuh.gov.tw; 2 Division of Thoracic Surgery, Department of Surgery, National Taiwan University Hospital, Taipei 10002, Taiwan; jangminglee@gmail.com; 3 Graduate Institute of Clinical Medicine, College of Medicine, National Taiwan University, Taipei 10051, Taiwan; 4 Division of Cardiovascular Surgery, Department of Surgery, National Taiwan University Hospital, Taipei 10002, Taiwan

**Keywords:** advanced esophageal cancer, aortic invasion, aortoesophageal fistula, salvage TEVAR, elective TEVAR

## Abstract

Aortoesophageal fistula (AEF) caused by esophageal cancer (EC) is a rare but life-threatening complication. However, the optimal management strategy remains undetermined. Previous cases have demonstrated that thoracic endovascular aortic repair (TEVAR) is effective for prophylactic management. In our study, we evaluated the management of AEF with elective TEVAR over salvage TEVAR. In our single-center retrospective cohort study, forty-seven patients with cT4M0 EC were included in this study, and we divided them into salvage (Group S) and elective (Group E) groups based on whether TEVAR was performed before the hemorrhagic AEF occurred. Our study outcomes included survival and complication rate after TEVAR. Group E showed better overall 90-day survival and aortic-event-free survival in 90-day and 180-day over Group S. More patients in Group E could receive subsequent chemoradiotherapy or esophagectomy. Significantly fewer AEF-related complications, including recurrent hemorrhagic events after TEVAR, hypoperfusion-related organ injury, and bloodstream infection, were noted in Group E. In patients with advanced EC-invading aorta, elective TEVAR offered an early overall and aortic-event-free survival benefit compared to salvage TEVAR. By reducing the AEF-related complications, elective TEVAR could provide more patients receiving subsequent curative-intent treatment.

## 1. Introduction

Aorto-esophageal fistula (AEF) is a rare and lethal entity, and the difficulty of making a diagnosis of AEF is well-known due to its representative symptom from sentinel hematemesis to massive hematemesis, and the symptom-free interval is unpredictable. Advanced esophageal cancer (EC) with aortic invasion is the third common cause of AEF, after thoracic aortic aneurysm and ingestion of a foreign body [1,2], owing to the anatomical proximity of the esophagus and aorta. Traditionally, advanced EC with aortic invasion is classified as T4b disease according to the 8th American Joint Committee on Cancer (AJCC) staging system [3] and is considered inoperable. However, there is growing evidence showing that radical surgical resection after chemoradiotherapy (CRT) can improve survival in selected patients [4,5,6,7]. Esophagectomy with combined open resection of the aorta has been performed in some Japanese high-volume centers, but the 30-day mortality was high [8,9].

Given the established advantage of endovascular thoracic aortic grafting (TEVAR) over the open aortic replacement for thoracic aortic aneurysm, several case series of esophagectomy after elective TEVAR have been reported. These findings indicated that the clinical benefit of TEVAR before esophagectomy provided better progression-free survival in patients with EC in terms of the intraoperative aortic hemorrhage and the achievement of R0 resection [4,8,10,11]. In cases with salvage TEVAR after the onset of AEF, though temporary hemostasis could be achieved, endograft infection (EGI) would inevitably occur due to direct contact of the inner lumen of the esophagus and the thoracic aorta. In these patients, the salvage TEVAR procedure was considered as a palliative therapy, and patients would end up with conservative treatment due to the unresectable nature of the advanced EC, which was almost invariably fatal [12,13,14,15,16]. As described in limited clinical experience, only a few reports analyzed the clinical value of elective TEVAR for patients with advanced EC with aortic invasion to prevent fatal complications of AEF and aortic hemorrhage during esophagectomy.

Our study aimed to compare the feasibility, safety, and clinical outcomes of elective TEVAR versus salvage TEVAR after the acute development of AEF in patients with advanced EC with T4b disease, stage IVA without distant metastasis. To our knowledge, this was currently the largest single-center retrospective cohort study on this subject.

## 2. Materials and Methods

### 2.1. Data Collection

This retrospective single-centered cohort study was approved by the Institutional Review Board of the Ethics Committee in National Taiwan University Hospital, and the requirement of informed consent was waived because of the retrospective nature of the study. This study was registered in ResearchRegistry, and the study has been reported according to the STROCSS guideline [17].

By reviewing the electronic medical records from March 2011 to March 2021 in National Taiwan University Hospital, patients with T4b EC of squamous cell type consulted for TEVAR were included in this study. Exclusion criteria included death due to hemorrhagic events before salvage TEVAR and distant metastatic EC. Data regarding independent prognostic factors for EC were collected [18,19]. The following patient information was recorded: sex, age when receiving TEVAR, tumor location, clinical stage of EC, treatment of EC before and after receiving TEVAR, and survival time after receiving TEVAR.

Information including the death registration was collected through the Integrated Medical Database in National Taiwan University Hospital and the National Health Insurance Research Database. The follow-up data were truncated on 30 June 2021. Patients were considered lost to follow-up if no information was available in any of the databases during follow-up.

### 2.2. Study Design

**Figure 1:**
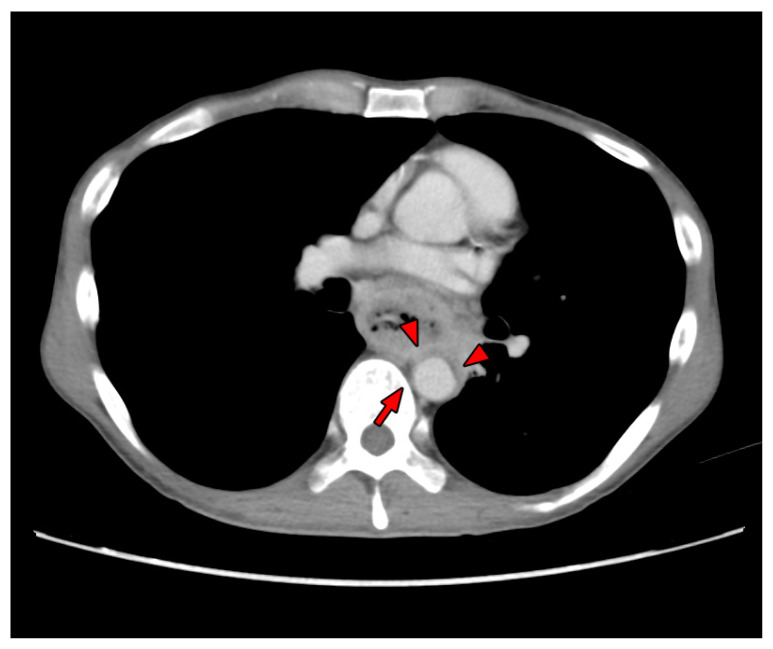
Computed tomography image of a patient with esophageal cancer before receiving elective thoracic endovascular aortic repair (TEVAR). The arrowheads are esophageal cancer invading the aortic wall. The arrow demonstrates the obliteration of the triangular fat space between the esophagus and thoracic aorta. These findings are indicated for elective TEVAR.

We divided patients into two groups: the salvage group (Group S), who received TEVAR after AEF-induced hemorrhagic events, and the elective group (Group E), who received TEVAR before any AEF-related hemorrhagic event was noted. A patient was considered the surgical candidate for elective TEVAR only if the staging computed tomography (CT) showed a T4b EC lesion without distant metastasis. The indication of TEVAR for patients in the elective group was either EC contacting greater than 90° of the descending thoracic aorta or obliteration of the triangular fat space between the esophagus and thoracic aorta on computed tomography (CT) or Positron emission tomography (PET), as indicated in Figure 1 [20,21]. 

### 2.3. TEVAR Procedure and Post-Procedural Management

All procedures were conducted under general anesthesia by using either femoral cutdown or percutaneous access in a hybrid suite (Artis zeego system, Siemens Healthcare, Forchheim, Germany). In Group E, the treatment goal was to cover the site of aortic invasion adjacent to the EC to prevent the aortic bleeding, while in Group S, this TEVAR procedure was performed to stop the acute hemorrhage from the AEF. The location of the invasive lesion or acute hemorrhage was determined by preoperative CT and PET scan, measuring proximally from the left subclavian artery (LSCA) or distally to the celiac artery. If the patient’s condition was stable before TEVAR, the oral edge of the tumor was marked with a radiopaque metallic clip by esophagoscopy to mark the optimal site for the stent graft. Both proximal and distal landing zones were selected between Zones 2 to 5 depending on the location of EC; at least 2 cm of the healthy aorta was required. The selected diameter size of the stent graft was 10–20% larger than the aortic diameter at the proximal landing zone. Tapered devices or telescopes with smaller devices were selectively used in patients with smaller distal aortic landing zones. A chimney procedure or physician-modified fenestration was performed as needed in some patients for LSCA revascularization to achieve an adequate proximal landing, but it was rarely performed for the distal landing zone. Routine spinal drainage was not required due to the short segment of aortic coverage or the emergent situation. Blood pressure was strictly controlled at 140/80 mmHg postoperatively to prevent spinal cord injury after TEVAR.

After the procedure, patients in the elective group were admitted to the intensive care unit (ICU) for 1 day to monitor hemodynamics and neurological status. The prophylactic antibiotic with intravenous first-generation cephalosporin or vancomycin was given. In salvage cases, due to the unstable perioperative hemodynamics, patients were sent to the ICU for further monitoring of hemodynamics until stabilization. Considering the AEF, broad-spectrum prophylactic antibiotics with third or fourth generation cephalosporin with or without metronidazole was given for coverage of enteric aerobic and anaerobic flora.

For elective patients, chemotherapy or radiotherapy generally starts 2–4 weeks after receiving TEVAR. In the salvage patients, due to the hemodynamic instability, the time of receiving CRT depends on the patient’s clinical condition.

### 2.4. Study Outcome

In our study, the primary outcomes included the freedom from all-cause mortality and aorta-related event-free survival analysis after TEVAR. The postoperative TEVAR secondary outcomes were the complications including recurrent or new aortic hemorrhagic events, EGI evidenced by the bloodstream infection with enteric commensals, reversible or irreversible hypoperfusion-related organ dysfunction, and neurological complications. The subsequent definitive CRT or salvage esophagectomy after TEVAR and the index length of hospital stay were also recorded.

### 2.5. Statistical Analysis

For statistical analysis, continuous variables between Groups S and P were compared using the Mann–Whitney U-test. The chi-square and Fisher’s exact tests were used to compare categorical variables. The log-rank test was used to analyze overall survival and aortic-related event-free survival between groups. Multiple regression analysis and the Cox regression model were applied to establish the independence between two groups. A *p*-value less than 0.05 was considered significant. All statistical analyses were performed using SPSS, version 25.0 (IBM, Armonk, NY, USA).

## 3. Results

**Figure 2:**
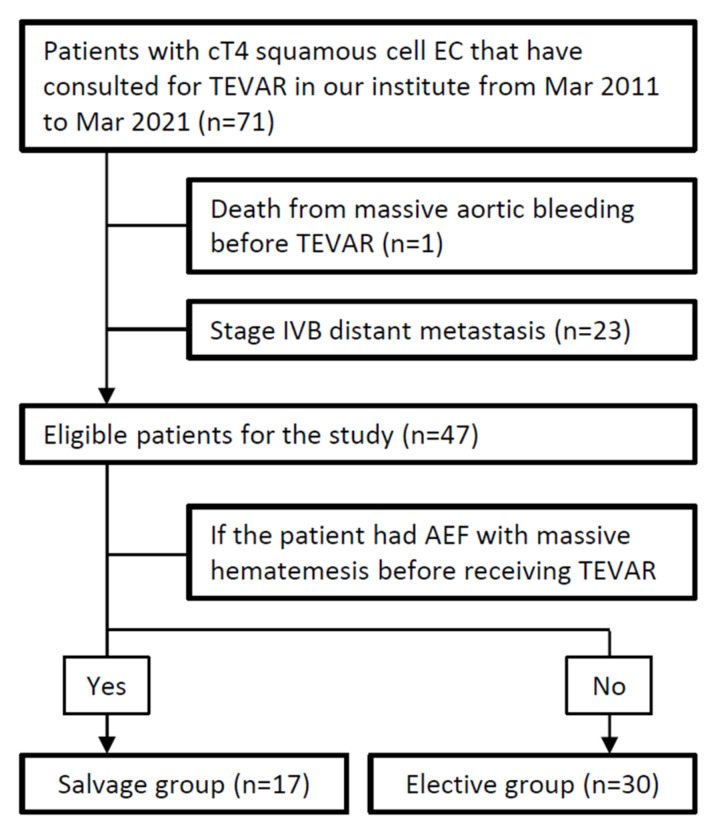
Flow chart of patient selection. (EC: esophageal cancer, TEVAR: thoracic endovascular aortic repair, AEF: aortoesophageal fistula).

A total of 71 patients were identified. Among these patients, 24 of them were excluded from our study because of death from massive aortic bleeding at the emergency department before the salvage TEVAR (n = 1) or because they were diagnosed with EC stage IVb with distant metastasis (n = 23). Consequently, the data of the 47 remaining patients were included in our analyses, with 17 patients in Group S and 30 patients in Group E. The flow chart of patient selection was presented in Figure 2.

### 3.1. Patient Characteristics

Table 1 presented the patients’ demographic information. The mean age of our study population was 59.10 ± 9.94 years, and the mean BMI was 20.80 ± 3.50 kg/m^2^. Before TEVAR, 12 (71%) of the patients received CRT, while two (12%) of the patients received esophagectomy in Group S, and six (20%) of the patients received CRT but none of the patients received esophagectomy in Group E.

### 3.2. Primary Outcomes

**Figure 3:**
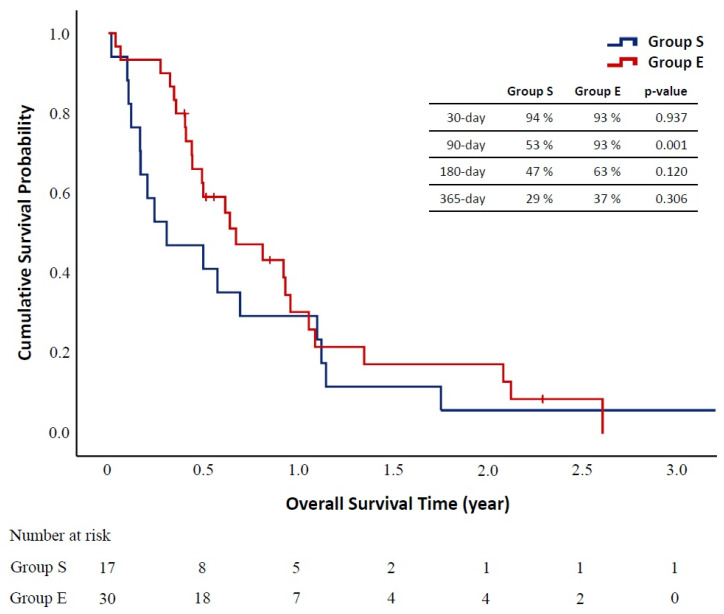
Kaplan–Meier survival analysis between salvage group (Group S) and elective group (Group E) at 30 days, 90 days, 180 ays, and 365 days after the thoracic endovascular aortic repair operation.

**Figure 4:**
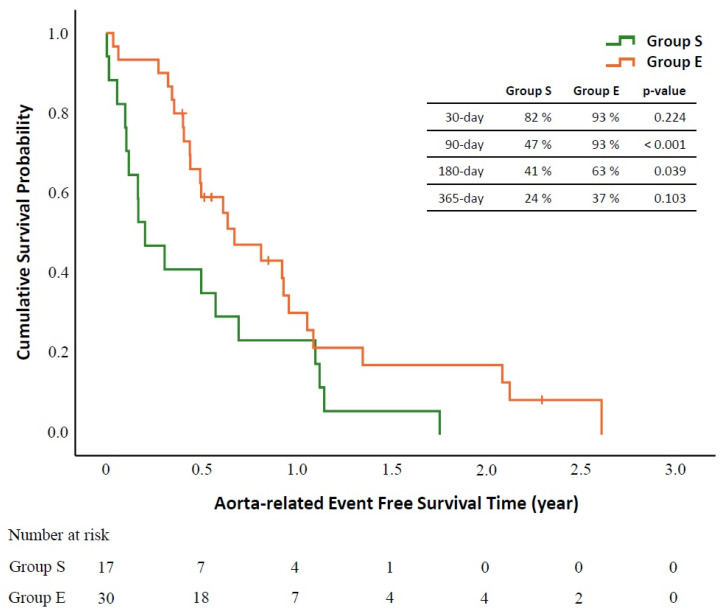
Kaplan–Meier aorta-related event-free survival analysis between salvage group (Group S) and elective group (Group E) at 30 days, 90 days, 180 days, and 365 days after the thoracic endovascular aortic repair operation.

The results of the overall survival analysis were shown in Figure 3. Until the end of follow-up on 30 June 2021, six patients remained alive (one in group S and five in Group E). Patients in Group E had significantly better overall 90-day survival rate compared to those in group S. No significant survival difference was observed in the 30-, 180-, and 365-day analysis. The result of aortic-related event-free survival analysis was shown in Figure 4. Two patients in Group S had aorta-related events manifested with recurrent aortic hemorrhage. One of them received thoracotomy for hemostasis, and the other one received palliative therapy. The aortic-related event-free survival analysis suggested that Group E had a better survival rate in 90 and 180 days.

Among all factors in multiple regression analysis of survival time, pre-TEVAR esophagectomy and alcohol statistically significantly predicted the length of survival time. The results of the statistical significance of each independent variable were listed in Supplementary Table S1. However, Supplementary Table S2 showed a Cox regression model suggesting that alcohol did not statistically significantly predict the overall 90-day survival, aorta-related event-free 90- and 180-day survival.

### 3.3. Secondary Outcomes

The perioperative details and secondary outcomes were summarized in Table 2. In Group S, two patients had aortic-related events manifested with recurrent AEF hemorrhage. Two patients had tumor bleeding despite receiving TEVAR, but their hemodynamics were stabilized with component therapy. Irreversible hypoperfusion-related multi-organ failure was noted in one patient, and reversible renal and hepatic dysfunction in the other two patients. Three patients were diagnosed with EGI evidenced by bloodstream infection of Fusobacterium nucleatum, Streptococcus constellatus, and Pseudomonas aeruginosa. In Group E, no patient experienced acute aortic bleeding after definitive CRT or esophagectomy after TEVAR. Only one patient developed transient hepatic dysfunction after TEVAR. The composite perioperative complication rate of Group S was statistically significantly higher than that of Group E (47% vs. 3%, *p* < 0.001). Patients in Group E had a statistically higher chance of receiving subsequent definitive CRT and salvage esophagectomy than Group S.

## 4. Discussion

In this study, elective TEVAR could prevent EC-associated AEF and more patients could undergo subsequent definitive CRT or salvage esophagectomy. In salvage TEVAR, though patients could achieve temporary hemostasis from the life-threatening AEF, it was associated with a poor 90-day overall survival, and 90-day to 180-day aorta-related event-free survival compared to elective TEVAR because of the increased periprocedural complications.

AEF was a life-threatening complication of advanced EC. It was more commonly seen in patients with advanced EC invading the aorta after concurrent CRT with a reported incidence of 10–29% [22]. In acute hemorrhage, salvage TEVAR has been reported to enable effective temporary hemostasis as a bridge to definite esophagectomy and open aortic grafting [12]. However, the acute hemodynamic instability from the aortic hemorrhage usually resulted in systemic hypoperfusion and subsequent multiple organ failure. In patients with advanced EC, salvage esophagectomy was usually associated with high morbidity and mortality rate due to the dense fibrotic scar after CRT, which made it difficult to identify the exact dissecting plane and possible intraoperative aortic injury. The AEF complicating EGI in the unresectable advanced EC was even considered unfit for subsequent definitive open surgical repair and carried the worst prognosis. As demonstrated in our study, patients undergoing salvage TEVAR had the higher recurrent aortic bleeding and EGI compared to none in the elective TEVAR. Because of the unexpected aortic bleeding with hypovolemic shock status, more patients experienced hypoperfusion-related multiple organ failure, which was correlated to poor 90-day all-cause mortality and 90–180 day aorta-related event-free survival. Nevertheless, patients in group S would have died at the onset of AEF or within a couple of days if they had not undergone TEVAR. Therefore, salvage TEVAR was still regarded as an effective life-saving procedure for these critical patients.

Traditionally, esophagectomy in EC patients with aortic invasion is considered contraindicated. According to the National Comprehensive Cancer Network guideline, the recommended treatment is definitive CRT without surgery. However, disease progression is frequently encountered after such treatment with a median survival of 10.6 months, and 14.6% of patients end up with AEF during or after treatment [2]. Instead of the conservative treatment, radical surgical resection after CRT has been suggested by some Japanese high-volume centers, including combined esophagectomy with aortic resection since early 2000. This treatment strategy could provide a chance of improved survival for selected patients. However, this open aortic grafting carried a high 30-day mortality rate of up to 9.62% [9]. TEVAR has been successfully performed in patients with thoracic aortic aneurysms or dissections for the last three decades. Given the advantages of the minimally invasive endovascular approach and lower incidence of periprocedural complications, the combined use of elective TEVAR for partial resection of the aortic wall to prevent fatal intraoperative bleeding and esophagectomy has been investigated. Although there have been several case series of esophagectomy after elective TEVAR, the efficacy of the strategy was currently not well-established. However, compared to salvage TEVAR, the clinical benefits of elective TEVAR not only provided the safety of subsequent esophagectomy and the achievement of R0 resection but also the long-term survival time in selected patients [1,8,23]. In this study, elective TEVAR was performed in relatively stable patients before the unexpected hemorrhagic events. It could enable further treatment of EC including CRT or esophagectomy from the prevention of hypoperfusion-related or recurrent bleeding complications including AEF-related or tumor bleeding, thus improving patient survival. elective TEVAR also constituted a barrier between the EC and thoracic aorta. Even after the tumor has disintegrated by the CRT, it could effectively prevent the occurrence of AEF and subsequent EGI. Our secondary outcome showed that the low perioperative TEVAR complications might deserve the prophylactic nature of this procedure.

In this study, elective TEVAR did not provide a long-term survival benefit in patients with T4b EC compared to salvage TEVAR. Elective TEVAR could improve short-term survival by preventing aortic-related mortality. However, the long-term survival still depended considerably on tumor progression, which was difficult to be dealt with elective TEVAR. In the previous literature, the combined use of TEVAR and esophagectomy could improve local tumor control and progression-free survival but not the overall survival [4]. However, many promising treatment modalities for locally advanced EC have emerged in recent years [5,24], including a Phase II trial conducting the induction chemotherapy with docetaxel, cisplatin, and 5-fluorouracil followed by esophagectomy for patients with locally advanced EC [24] and a Phase III trial comparing the induction chemotherapy strategy with definitive chemoradiotherapy. For patients with elective TEVAR, they might have a higher chance of receiving these novel induction treatments and curative-intent esophagectomy, since they have a higher survival rate at 90 days and a lower AEF-related complication rate caused by tumor necrosis from CRT. With these clinical trials, the long-term survival benefits of elective TEVAR might be anticipated in the future.

We acknowledged the limitation of statistical power under such a single-center retrospective study with a small patient population, although it was currently the largest cohort in the literature to compare elective and salvage TEVAR in T4b EC patients. Our study focused explicitly on squamous cell carcinoma of the esophagus; a few patients with other histopathological subtypes of EC, including adenocarcinoma, small-cell carcinoma, and undifferentiated carcinoma, were excluded. Thus, our results might not apply to other types of EC. The generalizability of our study might be limited to Southeast Asian cohorts. Further multi-center prospective studies to investigate whether the intervention with elective TEVAR could make a difference to survival for such patients were required.

## 5. Conclusions

In this study, elective TEVAR could offer an early 90-day survival benefit and 90- to 180-day aorta-related event-free survival over salvage TEVAR by reducing the AEF-related complications. It could also prevent EC-associated AEF and enable patients to receive subsequent definitive CRT or salvage esophagectomy for the curative-intent treatment. elective TEVAR appeared to be safe and effective and might become part of the routine approach before CRT or esophagectomy in patients with advanced EC with aortic invasion.

## Figures and Tables

**Table 1:** Patient demographic data in each group.

	Total (n = 47)	Salvage Group (n = 17)	Elective Group (n = 30)	*p*-Value
Age (years) *	61 (36–80)	57 (38–71)	62 (36–80)	0.249
BMI (kg/m^2^) *	20.7 (13.0–27.2)	20.7 (16.6–27.2)	20.7 (13.0–26.9)	0.603
Sex				0.294
Male	44 (94%)	15 (88%)	29 (97%)	
Female	3 (6%)	2 (12%)	1 (3%)	
ECOG				0.597
0	18 (38%)	4 (24%)	14 (47%)	
1	15 (32%)	6 (35%)	9 (30%)	
2	6 (13%)	3 (18%)	3 (10%)	
3	6 (13%)	3 (18%)	3 (10%)	
4	2 (4%)	1 (6%)	1 (3%)	
Substance use				
Alcohol	36 (77%)	12 (71%)	24 (80%)	0.349
Betel nut	18 (38%)	6 (35%)	12 (40%)	0.750
Cigarette	38 (81%)	13 (76%)	25 (83%)	0.417
Tumor Location				0.255
Upper Third	5 (11%)	3 (18%)	2 (7%)	
Upper and Middle Third	6 (13%)	4 (24%)	2 (7%)	
Middle Third	18 (38%)	5 (29%)	13 (43%)	
Middle and Lower Third	8 (17%)	3 (18%)	5 (17%)	
Lower Third	10 (21%)	2 (12%)	8 (27%)	
Comorbidity				
Coronary artery disease	3 (6%)	1 (6%)	2 (7%)	0.706
Chronic kidney disease	3 (6%)	2 (12%)	1 (3%)	0.294
Diabetes mellitus	5 (11%)	4 (24%)	1 (3%)	0.051
Hypertension	21 (45%)	6 (35%)	15 (50%)	0.253
Liver cirrhosis	7 (15%)	5 (29%)	2 (7%)	0.049
COPD	5 (11%)	1 (6%)	4 (13%)	0.397
Cerebrovascular accident	1 (2%)	0	1 (3%)	0.638
Others	7 (15%)	3 (18%)	4 (13%)	0.499
Pre-TEVAR treatment				
Chemoradiotherapy	18 (38%)	12 (71%)	6 (20%)	0.001
Esophagectomy	2 (4%)	2 (12%)	0	0.126

Data are presented as n (%) or mean ± stand deviation. * Non-parametric continuous data are presented as median (range). BMI: body mass index, ECOG: eastern cooperative oncology group performance status, TEVAR: thoracic endovascular aortic repair, COPD: chronic obstructive pulmonary disease.

**Table 2:** The perioperative details and secondary outcomes in each group.

	Total (n = 47)	Salvage Group (n = 17)	Elective Group (n = 30)	*p*-Value
Post-TEVAR Treatment				
Chemoradiotherapy	34 (72%)	9 (53%)	25 (83%)	0.025
Esophagectomy	22 (47%)	4 (24%)	18 (60%)	0.017
Post-TEVAR Complications	9 (19%)	8 (47%)	1 (3%)	<0.001
Recurrent AEF Hemorrhage	2 (4%)	2 (12%)	0	
Tumor bleeding	2 (4%)	2 (12%)	0	
Endograft Infection	3 (6%)	3 (18%)	0	
Hypoperfusion-related Irreversible Organ Failure	1 (2%)	1 (6%)	0	
Hypoperfusion-related Reversible Organ Dysfunction	3 (6%)	2 (12%)	1 (3%)	
Neurological Complication #	0	0	0	
Post-TEVAR Index Hospital Stay (days) *	12 (1–124)	16 (3–124)	11 (1–100)	0.061
Device				0.105
C-TAG^®^, Gore^®^	24 (51%)	12 (71%)	12 (40%)	
Valiant™, Medtronic	21 (45%)	5 (29%)	16 (53%)	
Zenith Alpha™, Cook	2 (4%)	0	2 (7%)	
Proximal Landing zone				0.005
Zone 2	5 (11%)	3 (18%)	2 (7%)	
Zone 3	19 (40%)	11 (65%)	8 (27%)	
Zone 4	23 (49%)	3 (18%)	20 (67%)	
TEVAR length (cm) *	15.0 (10.0–19.0)	15.0 (10.0–19.0)	15.0 (10.0–19.0)	0.653
TEVAR diameter				
Proximal (mm) *	31 (24–38)	31 (26–37)	31 (24–38)	0.712
Distal (mm) *	28 (21–38)	26 (21–34)	28 (24–38)	0.092
Concomitant procedure				
Total LSCA procedure	5 (11%)	3 (18%)	2 (7%)	0.336
LSCA revascularization	4 (4%)	2 (12%)	2 (7%)	
LSCA embolization	1 (2%)	1 (6%)	0	

Data are presented as n (%) or mean ± stand deviation. * Non-parametric continuous data are presented as median (range). EC: esophageal cancer, TEVAR: thoracic endovascular aortic repair, AEF: Aortoesophageal fistula, LSCA: Left subclavian artery. # Neurological complication including spinal cord injury and stroke.

## Data Availability

The datasets retrieved and analysed in this study are not publicly available due to patient privacy but are available from the corresponding author on reasonable request.

## Supplementary Materials

The following are available online at www.mdpi.com/article/10.3390/biomedicines9121889/s1, Table S1: Multiple regression analysis of the length of survival time, Table S2: Cox regression analysis of groups and using alcohol.
