# Supplementary material for: Comparison of Clinical Outcomes between Salvage and Elective Thoracic Endovascular Aortic Repair in Patients with Advanced Esophageal Cancer with Aortic Invasion: A Retrospective Cohort Study
Source: Biomedicines. 2021 Dec 12;9(12):1889. doi: 10.3390/biomedicines9121889 (PMC8698351; doi:10.3390/biomedicines9121889)
Supplement: Supplementary file 1 [file biomedicines-09-01889-s001.zip › biomedicines-1476461-supplementary.pdf]

Supplementary Table S1. Multiple regression analysis of the length of survival time. Factors including pre-TEVAR esophagectomy and alcohol statistically significantly predicted the length of survival time.

	Unstandardized Coefficients		Standardized Coefficients	T	p-value
	B	Standard Error			
Age	-5.389	6.683	-.151	-0.806	0.427
BMI	-9.292	17.646	-.092	-0.527	0.603
Sex	-18.241	260.413	-.013	-0.070	0.945
ECOG	-66.339	58.880	-.222	-1.127	0.270
Tumor Location	21.106	102.471	.037	0.206	0.838
Substance use					
Alcohol	-329.224	149.509	-.396	-2.202	0.036
Betel nut	111.361	135.820	.154	0.820	0.419
Cigarette	126.799	162.126	.142	0.782	0.441
Comorbidity					
Coronary artery disease	434.508	318.790	.302	1.363	0.184
Chronic kidney disease	141.465	254.529	.098	0.556	0.583
Diabetes mellitus	-157.580	195.089	-.138	-0.808	0.426
Hypertension	-33.723	126.508	-.048	-0.267	0.792
Liver cirrhosis	-125.027	174.623	-.127	-0.716	0.480
COPD	-12.941	180.470	-.011	-0.072	0.943
Cerebrovascular accident	-454.489	461.793	-.187	-0.984	0.334
Others	314.485	424.164	.129	0.741	0.465
Pre-TEVAR treatment					
Chemoradiotherapy	-84.780	125.941	-.117	-0.673	0.507
Esophagectomy	787.442	294.602	.452	2.673	0.013

BMI: body mass index, ECOG: eastern cooperative oncology group performance status, TEVAR: thoracic endovascular aortic repair, COPD: chronic obstructive pulmonary disease

Supplementary Table S2. Cox regression analysis of groups and using alcohol for the overall 90-day survival (2A), aorta-related event-free 90-day survival (2B), and aorta-related event-free 180-day survival (2C). Alcohol is not a statistically significantly predictor for these survival analysis.

(2A)

	Regression Coefficients	Standard Error	Odds Ratio (95% CI)	p-value
Groups (Elective vs. Salvage)	2.111	0.798	8.259	0.008
Substance use - Alcohol	-0.053	0.695	0.949	0.940

(2B)

	Regression Coefficients	Standard Error	Odds Ratio (95% CI)	p-value
Groups (Elective vs. Salvage)	2.435	0.778	11.420	0.002
Substance use - Alcohol	0.464	0.614	1.591	0.450

(2C)

	Regression Coefficients	Standard Error	Odds Ratio (95% CI)	p-value
Groups (Elective vs. Salvage)	1.041	0.435	2.833	0.017
Substance use - Alcohol	0.238	0.483	1.269	0.622
